# Affected Microcirculation and Vascular Hemodynamics in Takayasu Arteritis

**DOI:** 10.3389/fphys.2022.926940

**Published:** 2022-07-05

**Authors:** Christina Svensson, Niclas Bjarnegård, Per Eriksson, Hanna Jonasson, Tomas Strömberg, Christopher Sjöwall, Helene Zachrisson

**Affiliations:** ^1^ Department of Clinical Physiology, University Hospital, Linköping, Sweden; ^2^ Department of Health, Medicine and Caring Sciences, Division of Diagnostics and Specialist Medicine, Linköping University, Linköping, Sweden; ^3^ Department of Biomedical and Clinical Sciences, Division of Inflammation and Infection, Linköping University, Linköping, Sweden; ^4^ Department of Biomedical Engineering, Linköping University, Linköping, Sweden

**Keywords:** takayasu arteritis, microcirculation, intima-media thickness, augmentation index, peak oxygen saturation, breath hold index, ultrasound

## Abstract

**Introduction:** Takayasu arteritis (TAK) is a rare inflammatory disease affecting aorta and its major branches. Ultrasound (US) can detect inflammatory features in the arterial wall, but less is known regarding skin microcirculation and vascular hemodynamics. The aim was to study if assessment of these variables could add valuable information regarding vascular affection in TAK.

**Methods:** 17 patients diagnosed with TAK and 17 age- and sex-matched healthy controls were included. Microcirculatory peak oxygen saturation (OxyP) in the skin after induced ischemia was evaluated with laser Doppler flowmetry/diffuse reflectance spectroscopy. Cerebrovascular reserve capacity (CVR) in the brain was assessed with transcranial Doppler (TCD). Pulse waves were recorded in the radial artery by the aid of applanation tonometry, for calculation of central augmentation index (AIx75). Intima-media thickness (IMT) and stenosis/occlusions were evaluated using US in carotid and central arteries.

**Results:** Reduced OxyP (79 ± 8% vs. 87 ± 4%, *p* < 0.001) was seen in patients with TAK regardless of significant arterial stenosis/occlusion or not. Increased AIx75 (22.3 ± 13.6 vs. 9.2 ± 16.3, *p* = 0.01) was seen in TAK patients without significant stenosis/occlusions. No differences were found in CVR, regardless of proximal stenosis. However, signs of a more high-resistance flow profile were seen in arteria cerebri media.

**Conclusion:** Regardless of arterial stenosis or not, impaired microcirculation of the skin and preserved CVR in the brain were found in subjects with TAK. Signs of increased arterial stiffness in the brain and central arteries were observed. The value of these findings for prediction of future cardiovascular events needs to be clarified in further studies.

## Introduction

Takayasu arteritis (TAK) is a rare large vessel vasculitis primarily affecting aorta and its main branches, and the disease usually has its onset in young females. TAK has a worldwide distribution, although it is most common in the Asian population (Onen and Akkoc, 2017). The incidence of TA in Sweden is comparable to recently reported rates from other European studies, with an incidence rate estimated to 0.7/million inhabitants (Mohammad and Mandl, 2015).

Thickening of the vessel walls may result in stenosis and/or occlusion reported in up to 90% of the patients (Tombetti and Mason, 2019). Patients may develop ischemic symptom from specific organs due to restricted regional blood flow, and in addition often unspecific inflammatory symptoms like fever, weight loss and fatigue (Noel et al., 2013).

Ultrasound (US) with measurement of intima-media thickness (IMT) in carotid and central arteries is a reliable and validated imaging modality often used for both diagnose and follow up of TAK (Schäfer et al., 2020; Svensson et al., 2020a). However, less is known regarding skin microcirculation and vascular hemodynamics.

Microcirculation could be evaluated with different methods. Herein, we employed a new fiber-optic system that combines laser Doppler flowmetry (LDF) and diffuse reflectance spectroscopy (DRS) (Fredriksson et al., 2013; Jonasson et al., 2015). The system is easily accessible and non-invasive. In combination with various provocations, such as post-occlusive reactive hyperemia (PORH) test, it can measure microvascular reactivity in the forearm skin. The system estimates microcirculatory red blood cell tissue fraction, speed resolved perfusion, and oxygen saturation.

Higher central arterial wall stiffness is an independent risk marker for future cardiovascular events (Nürnberger et al., 2002). Indirect measurements of arterial stiffness can be evaluated with non-invasive techniques such as tonometry, where the tonometer is placed over the radial artery for calculation of augmentation index (AIx). Several papers have reported AIx to be an easy and reproducible parameter for indirect assessment of arterial stiffness. AIx is strongly correlated to direct measurements of arterial distensibility and can thereby be used as a surrogate for arterial stiffness (Ng et al., 2006; Vlachopoulos et al., 2010; Takemoto et al., 2021).

Transcranial Doppler (TCD) is a non-invasive method to study intracranial flow velocity and cerebrovascular reserve capacity (CVR). CVR represents the ability of the cerebral arteries to dilate and constrict in response to stimuli. Breath-holding can be used as a vasodilator stimulus, and breath hold index (BHI) is calculated based on the mean flow of the middle cerebral artery (MCA) before and after 30 s of breath holding (Khorvash et al., 2013). BHI correlates with other methods evaluating CVR, such as vasodilatation with acetazolamide (Müller et al., 1995).

The aims of our study were to evaluate vascular hemodynamics, including microvascular function in the skin and brain in patients with TAK, and furthermore, to examine the influence of significant stenosis or occlusion on microcirculatory parameters. We hypothesized that addition of these methods to a standard US of carotid and central arteries, could provide additional valuable information on vascular status in subjects with TAK.

## Material and Methods

### Subjects

In this cross-sectional study we included 17 patients (14 women, three men; mean age 41.7 ± 14.5 years) diagnosed with TAK based on the American College of Rheumatology (ACR) classification criteria (Arend et al., 1990). Computed tomography angiography (CTA) or Magnetic resonance imaging (MRI) angiography had been used for diagnosis. US showed inflammatory vessel wall changes in the aortic branches, representative of TAK in all patients (Supplementary Table S1). All patients were assessed as clinically stable when included. For each patient, the following data were recorded: height, weight, waist circumference, sagittal abdominal diameter and smoking habits. Variables concerning ongoing pharmacotherapy, and earlier cardiovascular events were collected by medical records. Blood pressure was determined with oscillometric technique in both arms (Dinamap PRO 200 Monitor, Criticon, Tampa, FL, United States ).

Seventeen healthy age- and sex-matched, non-medicated (except for contraceptives) controls without clinical signs of inflammatory or atherosclerotic disease (14 women, three men; mean age 41.1 ± 12.9 years), were examined using the same protocol as for the patients.

### Examination Procedure

All participants were asked to refrain from coffee or nicotine use 4 h prior to the measurements. The participants were acclimatized in a room with a temperature of 25 °C and dimmed lighting. The subjects had to rest for 15 min before start of the examination. A standardized examination procedure was used in all individuals. The same vascular sonographer performed all examinations and offline measurements.

### Laboratory Measurements

Standard cardiovascular and inflammatory laboratory tests were collected after 12 h overnight fasting including, total cholesterol, high-density lipoprotein (HDL), non-HDL cholesterol, Interleukin-6 (IL-6), and C-reactive protein (CRP) analyzed with high sensitive technique in plasma (Sjöwall et al., 2004; Svensson et al., 2020b; Enocsson et al., 2021).

### Microcirculation

**FIGURE 1:**
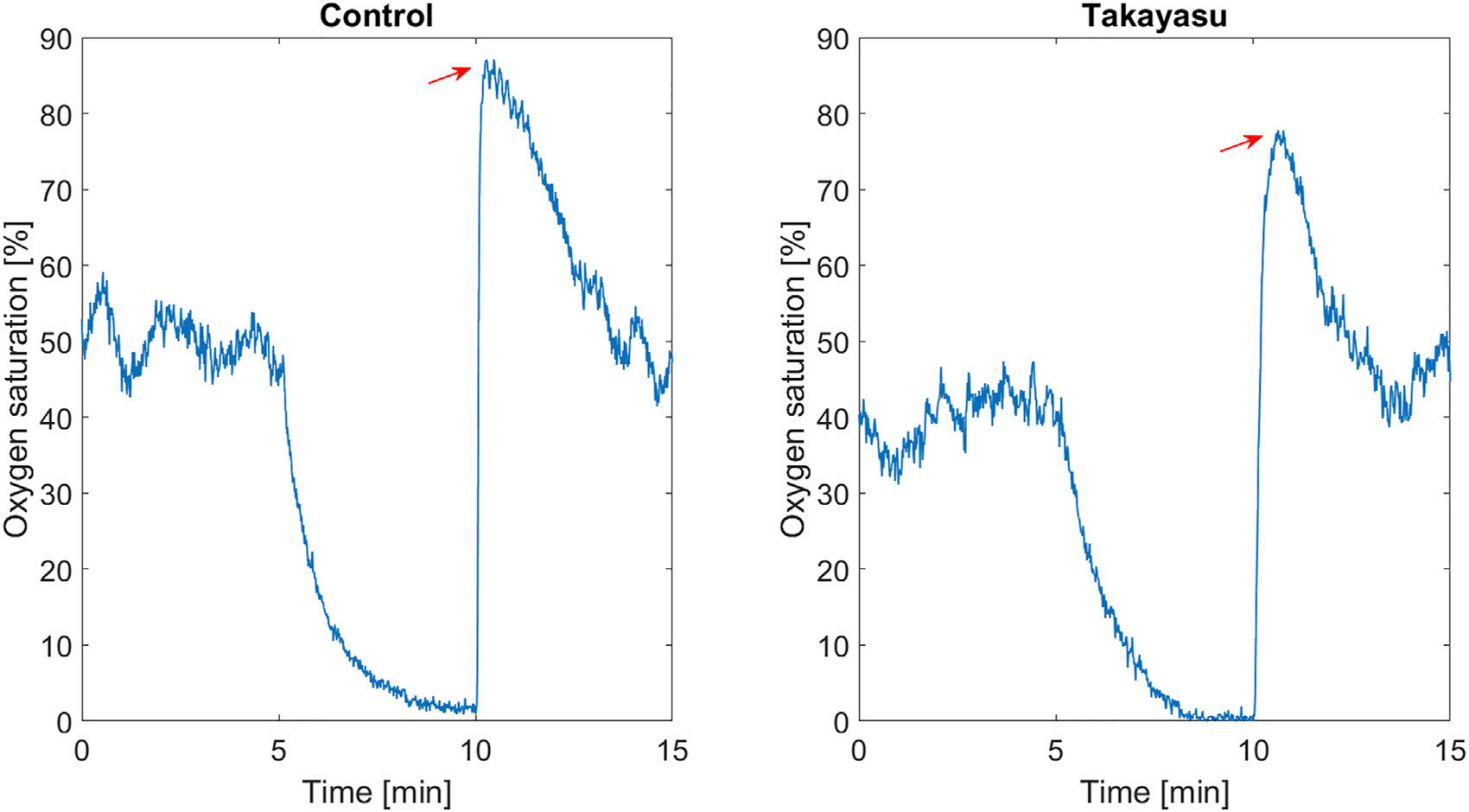
Oxygen saturation (%), baseline, during arterial occlusion (between 5 and 10 min), and in the post-occlusive hyperemia phase, in one healthy control and in one Takayasu patients with subclavian stenosis. Red arrow marks oxygen saturation peak.

The measurements were performed with PeriFlux 6000 EPOS system (Enhanced Perfusion and Oxygen Saturation, Perimed, Järfälla, Sweden). A sphygmomanometer cuff was placed on the upper arm. The EPOS probe was attached with double-adhesive tape on a visible vein free area on the forearm, approximately 10 cm below the cuff. A baseline measurement period of 5 min was followed by a 5‐minute suprasystolic occlusion of the upper arm ending with a 5‐minute post-ischemic measurement. After release of the cuff, PORH peak value of oxygen saturation (OxyP) was assessed (Figure 1). OxyP reflects overall microcirculatory function associated with vasodilator capacity and is better than perfusion values to discriminate between diseased patients and healthy controls and was thereby selected as the most robust value to report (Jonasson et al., 2019; Jonasson et al., 2020). Both arms were examined in the patient group. One arm was examined in the control group.

### Pulse Wave Analysis

**FIGURE 2:**
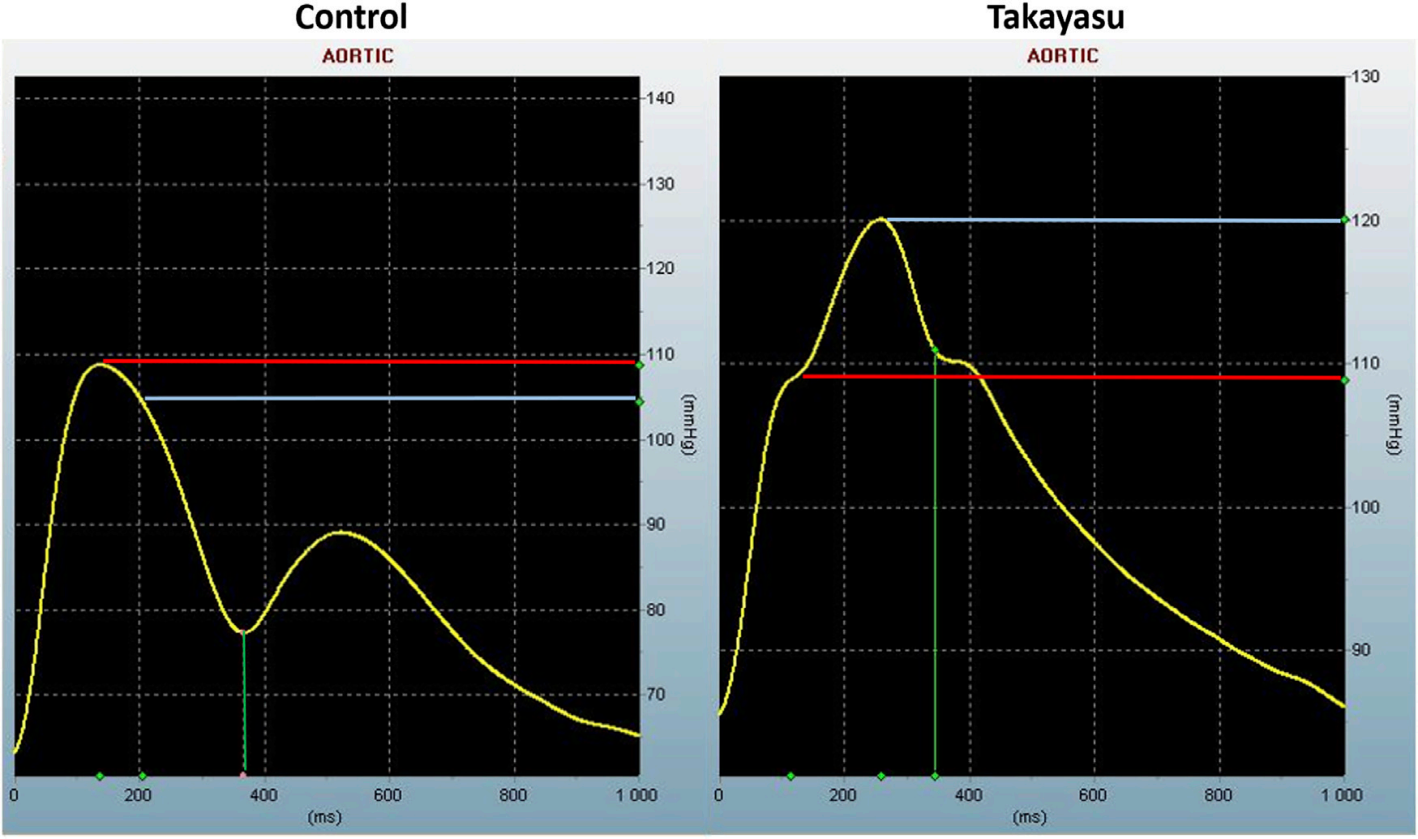
Aortic pulse wave analysis. Pulse pressure waveform from one of the healthy controls demonstrating reflecting wave during late systole, and from a Takayasu patient without proximal stenosis, where reflecting wave during early systole produces an augmented systolic pressure. The difference between red and blue line is the augmented pressure (the difference between the second and first systolic peak pressure). mmHg, millimeter of mercury; ms, millisecond.

Pulse wave analysis (PWA) was performed with applanation tonometry (SphygmoCor^®^ system, model MM3, AtCor Medical, Sydney, Australia) in both arms in TAK and in the right arm in controls. The procedure of the AIx measurements has been described previously (Svensson et al., 2021). Augmentation index adjusted to heart rate 75 (AIx75) was calculated. AIx is defined as [(Difference between the second and first systolic peak pressure)/Pulse Pressure] x100 (Figure 2). AIx denotes the relative aortic pulse pressure amplification in late systole from reflection waves.

### Transcranial Doppler

**FIGURE 3:**
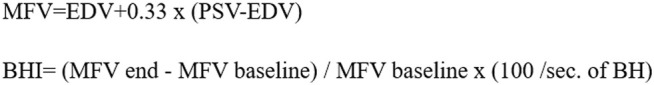
Equation of Mean flow velocity and Breath Hold Index. MFV, Mean flow velocity; EDV, End diastolic velocity; PSV, Peak systolic velocity; BHI, Breath hold index; sec, seconds; BH. Breath hold.

For evaluation of CVR the SONARA TCD system was used (SONARA Viasys TCD Machine, Healthcare solutions, Cardinal Health, Madison, WI 53711). Baseline and post breath-hold assessment of bilateral MCA were conducted using a 2 MHz probe. In the patient group, MCA was insonated bilaterally from the transtemporal window at depths of 45–55 mm with the prime focus on the M1 segment of MCA. In the control group only one side was evaluated. Normal inspiratory-expiratory cycles were allowed before each test. The test was performed at least twice with a period of normalized flow velocity between the measurements. The following variables were recorded during baseline measurements and after 30 s of breath hold: Peak systolic velocity (*PSV*) and end diastolic velocity (*EDV*). Mean Flow Velocities (MFV) and BHI were calculated offline (Figure 3).

### Ultrasound

For the IMT measurements, a GE Logic E10 US system (LOGIQ E10 XDclear 2.0 General Electric Medical Systems US, Wauwatosa, WI, United States ) with linear transducer L2-9 MHz was used. IMT was measured in the common carotid artery (CCA), internal carotid artery (ICA), subclavian artery (ScA), axillar artery (AxA), the brachiocephalic trunc and the aortic arch. Both sides were investigated. The procedure and evaluation of vessel wall characteristics has been described previously (Svensson et al., 2020b). In this paper we focused on IMT and occurrence of stenosis or occlusion. An atherosclerotic plaque was defined as a heterogeneous or calcified focal change in the vessel wall, or an increase of IMT of either 0.5 mm or 50% compared to the IMT in the adjacent wall.

Maximum systolic flow velocity was measured in all vessels to evaluate possible arterial stenosis. A significant stenosis (>50%) was defined as an increase in maximal systolic velocity (at least 2-fold increase) with narrowed vessel lumen and post stenotic flow pattern distally. Occlusion was defined as a vessel without any signs of color flow or detectable Doppler signals. For stenosis in the internal carotid artery (ICA), the ECST grading was used (Author Anonymous, 1998).

### Statistical Methods

OxyP, AIx75, BHI and IMT are presented as mean ± SD. Differences between the whole TAK group and controls, as well as TAK with or without stenosis/occlusion and controls were calculated using Student’s t-test. Pearson’s correlation test, as well as univariate linear regression were used to test any relationship between OxyP, AIx75 and BHI and each of the variables in Table 1, IMT values in Table 2 and occurrence of plaque. Multivariate linear regression was used to examine factors explaining OxyP, AIx75 and BHI. All variables significant in the univariate model were combined and a stepwise procedure was performed eliminating non-significant (*p* ≥ 0.05) variables until a multiple model with only significant variables remained. For missing data, no imputation analysis was performed. The levels of IL-6 were below the detection limit (1.5 ng/L) in six patients (35%), why this variable was handled as a categorical variable and analyzed with Pearson’s Chi-square test. Statistical analyses were performed using SPSS version 25.0 (IBM, Armonk, NY United States ).

**TABLE 1:** Detailed characteristics of included patients and controls presented as mean ± SD or n (%).

	**TAK**	**Controls**
	***n* = 17**	***n* = 17**
***Background variables***		
Age at examination (years)	41.7 ± 14.5	41.1 ± 13.0
Female gender, *n* (%)	14 (82)	14(82)
Disease duration, years (range)	12.4 ± 12.6 (0-40)	N/A
***Traditional risk factors and laboratory data***		
Body mass index (BMI) (kg/m²)	27.6 ± 4.5	25.5 ± 4.7
Waist circumference (cm)	97.5 ± 15.4	89.8 ± 14.1
Sagittal abdominal diameter (cm)	24.1 ± 4.7*	20.2 ± 3.8
Ever smoker (former or current), *n* (%)	2 (12)	0
Systolic blood pressure (mm Hg), Non stenotic arm	132 ± 17**	118 ± 16
Diastolic blood pressure (mm Hg), Non stenotic arm	73 ± 11	69 ± 8
Diabetes mellitus, *n* (%)	1 (6)	0
Total cholesterol (mmol/L)	5.0 ± 1.1	4.6 ± 1.1
High-density lipoprotein (HDL) (mmol/L)	1.5 ± 0.5	1.5 ± 0.3
Non HDL cholesterol (mmol/L)	3.5 ± 1.0	3.1 ± 1.0
High-sensitivity CRP (mg/L)	5.1 ± 7.2	2.1 ± 2.5
IL-6 (ng/L)	7.8 ± 11.7*	1.5 ± 01.1
***Medical treatment, ongoing***		
Glucocorticoid therapy *n* (%)	11 (65)	0
Mean daily Prednisolone dose (mg)	4.1	0
Antihypertensives, *n* (%)	10 (59)	0
Beta-blockers, *n* (%)	7 (41)	0
ARB/ACE inhibitors, *n* (%)	4 (24)	0
Other antihypertensives, *n* (%)	6 (35)	0
Warfarin, *n* (%)	4 (24)	0
Antiplatelet, *n* (%)	7 (41)	0
Statin therapy *n* (%)	2 (12)	0
Methotrexate, *n* (%)	1 (6)	0
Infliximab, *n* (%)	1 (6)	0
Certolizumab pegol, *n* (%)	1 (6)	0
Baricitinib, *n* (%)	3 (18)	0
Tocilizumab, *n* (%)	2 (12)	0
***Earlier cardiovascular events***		
Stroke/TIA, *n* (%)	1 (6)	0
Vascular intervention /surgery, *n* (%)	3 (18)	0
Angina/Infarction, *n* (%)	2 (12)	0
Aortic valve replacement, *n* (%)	3 (18)	0
PCI or CABG, *n* (%)	2 (12)	0

ACE, angiotensin converting enzyme; ARB, angiotensin II, receptor blocker; CABG, coronary artery bypass graft; CRP, C-reactive protein; N/A, not applicable; TAK, takayasu arteritis; TIA, transient ischemic attack; TNF, tumor necrosis factor; IL-6, Interleukin six; PCI, percutaneous coronary intervention.

*p < 0.05, **p < 0.01.

**TABLE 2:** Assessable arteries refers to arteries without occlusion or significant stenosis. Intima-media thickness (IMT), in measured areas. TAK, Takayasu arteritis; mm, millimeter; CCA, common carotid artery; ICA, internal carotid artery; SCA, subclavian artery; AxA, axillary artery; nd, not done; n, number of patients.

	**TAK**	**Controls**	**TAK**
	**(*n* = 17)**	**(*n* = 17)**	**Assessable IMT**
	**mean ± SD**	**mean ± SD**	**n (%)**
***Vessel***	***IMT*** (***mm***)	***IMT*** (***mm***)	
CCA right	1.03 ± 0.41***	0.52 ± 0.11	17 (100)
CCA left	0.96 ± 0.68*	0.51 ± 0.12	17 (100)
ICA right	0.55 ± 0.27	0.45 ± 0.13	16 (94)
ICA left	0.68 ± 0.41	0.46 ± 0.13	17 (100)
SCA right	0.66 ± 0.58	0.58 ± 0.14	13 (76)
SCA left	0.51 ± 0.47	0.47 ± 0.10	12 (71)
AxA right	0.62 ± 0.35	0.49 ± 0.11	15 (88)
AxA left	0.38 ± 0.32	0.44 ± 0.09	11 (65)
Brachiocephalic trunc	1.53 ± 0.91	nd	14 (82)
Aortic arch	1.08 ± 0.84	0.86 ± 0.35	13 (76)

p < 0.05*, p < 0.001***.

### Ethics Considerations

Oral and written informed consent was obtained from all patients and healthy controls. The study protocol was performed according to the Declaration of Helsinki and approved by the Regional Ethics Board in Linköping (Decision Nr. 2013/33–31 and 2017/572–32).

## Results

Basic demographics, laboratory data and ongoing medical therapies are shown in detail in Table 1. No significant differences were observed between TAK and controls except for sagittal abdominal diameter, systolic blood pressure and IL-6 -levels.

### Microcirculation

**FIGURE 4:**
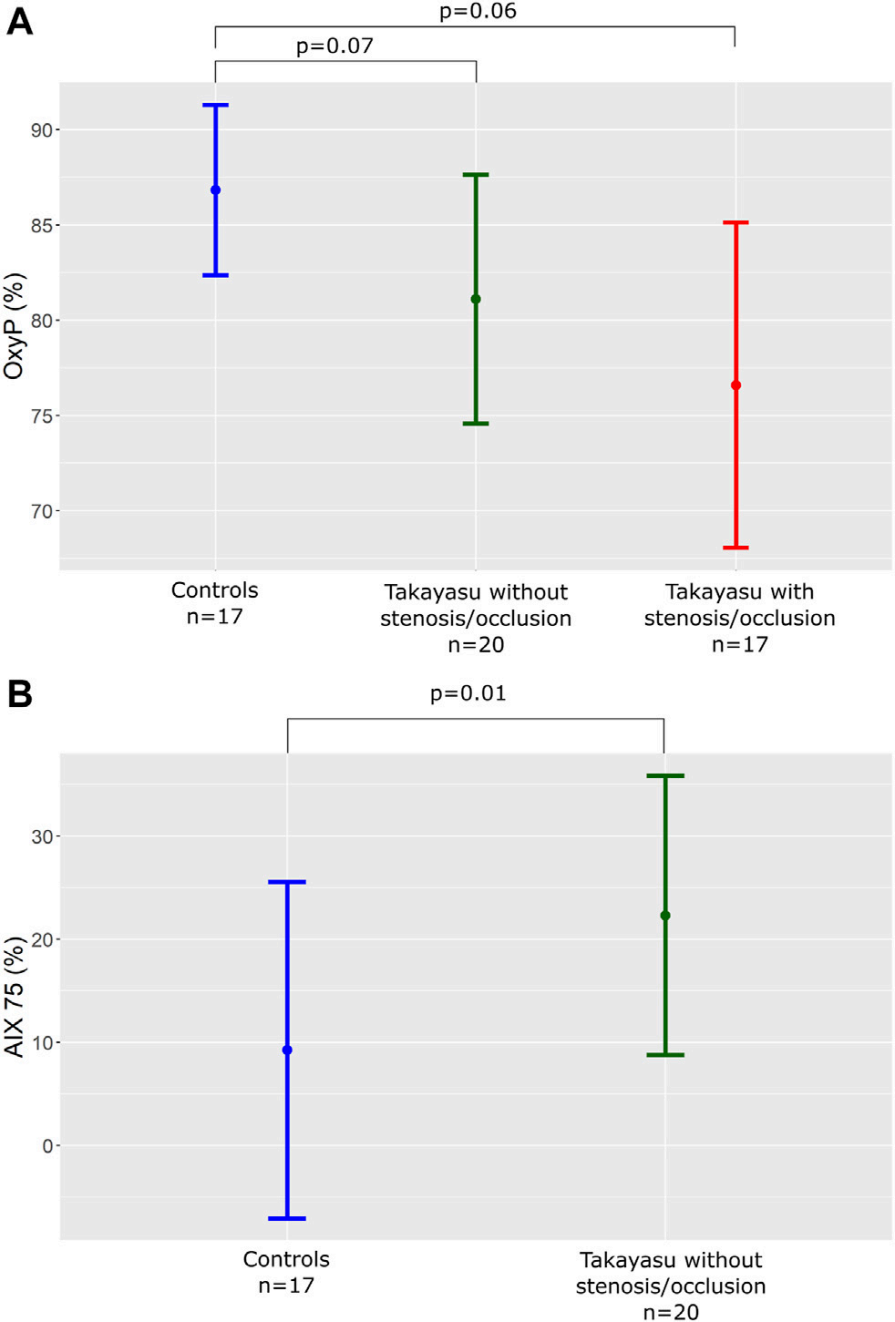
**(A)** OxyP (%) in controls and patients with or without significant stenosis or occlusion in the arm arteries. OxyP, peak oxygen saturation. **(B)** AIx75 (%) in controls and patients without significant stenosis or occlusion in the arm arteries. AIx75, Augmentation Index adjusted for heart rate 75.

Thirty-three measurements were performed in the patient group, and one test was interrupted due to arm pain. Seventeen measurements were performed in the control group. OxyP of the entire TAK group was significantly decreased compared to controls 79 ± 8% vs. 87 ± 4% (*p* < 0.001). TAK with stenosis had the lowest values (77 ± 9%) vs. TAK without stenosis (81 ± 7%) (*p* = 0.1; Figure 4A).

### Pulse Wave Analysis

AIx75 in TAK patients without stenosis/occlusions in upstream arteries, proximal to the measuring point (n = 20) was significantly increased compared to controls (*n* = 17), 22.3 ± 13.6 vs. 9.2 ± 16.3 (*p* = 0.01; Figure 4B). Arms with stenosis/occlusions were not possible to evaluate with peripheral tonometry.

### Transcranial Doppler

Twenty-eight BHI measurements were performed in the patient group. One patient had no acoustic window of the temporal bone, and was thereby excluded. In four patients, measurements were only possible to do on one side. BHI did not differ between TAK and controls 1.30 ± 0.10 vs. 1.41 ± 0.42 (*p* = 0.4). BHI did not differ regardless of significant carotid or central arteries stenosis/occlusion or not. BHI values in the two TAK patients with bilateral carotid stenosis/occlusion were 1.54 ± 0.33 and 0.61 ± 0.55 respectively.

Basic systolic velocity in MCA was not different between TAK and controls (0.90 ± 0.30 vs. 1.02 ± 0.14 m/s (*p* = 0.1)), whereas peak systolic velocity after breath holding differed (0.98 ± 0.51 vs. 1.29 ± 0.20 m/s (*p* = 0.02)). Both basic and peak diastolic velocity differed significantly (basic 0.32 ± 0.18 vs. 0.46 ± 0.07 m/s (*p* = 0.002), peak 0.47 ± 0.27 vs. 0.68 ± 0.10 m/s (*p* = 0.003)). Thus, in TAK a higher cerebral vascular resistance was found.

### Ultrasound

Compared to controls a significant difference in IMT was seen in CCA bilaterally (right 1.03 ± 0.41 vs. 0.52 ± 0.11 p=<0.001; left 0.96 ± 0.68 vs. 0.51 ± 0.12 *p* = 0.02). IMT values in other arteries and number of assessable IMT are shown in Table 2. Stenosis and occlusions are shown in Table 3. Eight patients had small atherosclerotic plaques, not affecting flow velocity. None of the controls had atherosclerotic plaques.

**TABLE 3:** Occurrence of significant stenosis and occlusions important for judgement of cerebral circulation (CCA, ICA) and brachial circulation (ScA, Axa, Brachiocephalic trunc).

TAK (*n* = 17)	*n* (%)	*n* (%)	*n* (%)
***Vessel***	***Right unilateral***	***Left unilateral***	***Bilateral***
CCA/ICA	2 (12)	3(18)	2 (12)
ScA/AxA/Brachiocephalic trunc	2 (12)	2 (12)	5 (29)

Relation of OxyP, AIx75 and BHI to background variables, traditional risk factor, medical treatment and IMT.

Microcirculatory, as well as central and cerebral hemodynamic values were unrelated to disease duration as well as standard cardiovascular and inflammatory laboratory tests. Three patients showed elevated hsCRP-levels above 10 mg/L.

In the univariate analysis of OxyP, hypertension treatment (other than beta-blocker or angiotensin II receptor blocker (ARB)/angiotensin-converting enzyme (ACE)) (B = -13.2, *p* = 0.05), warfarin treatment (B = -15.9, *p* = 0.03) and statin therapy (B = -29.9, *p* = 0.001), were negatively related to OxyP. However, in the multivariate analysis, only statin therapy remained negatively associated (B = -28.5, *p* = 0.01).

AIx75 and BHI did not correlate to treatment or any other background factors (Table 1).

A significant negative correlation was seen between OxyP and plaque occurrence (R = -0.56, *p* = 0.001). No significant correlations were seen between IMT in different vascular areas and OxyP, AIx75 or BHI values.

## Discussion

In patients with TAK we found impaired microcirculation assessed as peak oxygen saturation (OxyP) in the forearm, impaired peripheral wave reflections assessed as AIx75, and preserved cerebrovascular hemodynamics assessed as BHI. Increased arterial stiffness in the brain was also seen.

Rare clinical manifestations of impaired microcirculation in TAK have previously been described by Noel et al., i.e., occlusion of small retinal vessels, myocarditis, and necrotizing cutaneous vasculitis (Noel et al., 2013). However, the focus of this study is microcirculatory and vascular hemodynamics that potentially could be risk factors for cardiovascular disease.

For evaluation of microcirculation in the forearm we used a fiber-optic method combining measurement of microcirculatory blood flow and local oxygen saturation in the skin (Fredriksson et al., 2013; Jonasson et al., 2015). The system has been used to study microcirculatory perfusion in the Swedish Cardiopulmonary bioimage Study (SCAPIS), a large population-based cohort of men and women aged 50–65 years (Jonasson et al., 2020). The method can differ between normal and disturbed microcirculatory flow in the skin (Jonasson et al., 2017; Svensson et al., 2021; Jonasson et al., 2022), and the method has previously not been used in TAK.

Jonasson et al. has shown that age and sex are important variables to consider in evaluation of OxyP. They also demonstrated decreased OxyP values in patients with diabetes, hypertension and hyperlipidemia compared to patients without these diseases (Jonasson et al., 2020). Some of our TAK patients were treated for hypertension, and mean blood pressure was somewhat higher compared to controls. In the univariate analysis, antihypertensive drugs other than beta blockers or ARB/ACE inhibitors influenced OxyP, but the effect disappeared in multivariate analysis. In the appendix of the SCAPIS study, the lower OxyP values of patients with hypertension are shown (Jonasson et al., 2020). The OxyP values of our TAK population were much lower indicating that blood pressure alone cannot explain the decreased OxyP, although hypertension may have influenced OxyP to some degree. Our study showed no significant correlation between OxyP versus age or sex, but a significant negative correlation with plaque occurrence was seen. In the multivariate analysis, only statin therapy remained negatively associated. Reduction of plasma cholesterol by statins improves endothelial function and limit atherosclerosis (Wolfrum et al., 2003). However, our patient group is small and does not allow conclusions regarding influence of epidemiological factors on OxyP.

In this study we have not investigated potential differences in skin microcirculation between dominant and non-dominant arm. However, Leslie et al. did not find any significant differences in skin microcirculation response after intradermal saline injection stimulus, between dominant and non-dominant arm (Leslie et al., 2003). In Jonasson et al., post-occlusive peak values were studied in a large cohort consisting of 1765 study subjects. In those studies, the right arm was chosen for all study subject for consistency regardless of whether this was the dominant arm or not (Jonasson et al., 2020; Jonasson et al., 2022).

Endothelial function is assessed by measuring changes in vasomotor tone in response to various provocations where PORH is a proven model. Hypoxia stimulates vasodilation by different mechanism, such as increased nitric oxide release from endothelial cells (Musz et al., 2021). Endothelial dysfunction and arterial stiffness represent different aspect of vascular disease, but an interconnection between these pathophysiological processes is likely (Anderson, 2006). Vascular endothelium plays a decisive role in vascular tone and accordingly in arterial stiffness (Lerman and Zeiher, 2005; Duprez, 2010).

Alibaz-Oner et al. detected a decreased flow-mediated dilation (FMD) and increased IMT in TAK using US for IMT measurements and nitrate-induced dilatation for measurements of FMD (Alibaz-Oner et al., 2014). Using FMD, Rammos et al. showed reduced endothelial function in peripheral atherosclerotic disease, and improvement after treatment with drug-coated balloon or stent (Rammos et al., 2021). As shown in table 3, nine of our patients had unilateral or bilateral stenosis or occlusion in subclavian artery, axillary artery or the brachiocephalic trunc, and they had the lowest OxyP values. However, we observed impaired OxyP in the skin of forearms also in patients without significant stenosis or occlusion, implying that microcirculation in TAK is affected regardless of upstream large artery involvement or not. Microcirculation studies of TAK are scarce.

Herein, we observed impaired OxyP in the skin of forearms also in patients without significant stenosis or occlusion, implying that microcirculation in TAK is affected regardless of upstream large artery involvement or not. Microcirculation studies of TAK are scarce.

Increased arterial stiffness is a well-known risk factor of cardiovascular mortality (Vlachopoulos et al., 2010). Independent studies have shown that central arterial stiffness is increased in older individuals and in different cardiovascular diseases (Nichols and Singh, 2002). In this study we found increased AIx75 in TAK patients without significant stenoses/occlusions. However no significant correlation with age was seen, a potential explanation of this is the small size of the patient group.

Ng et al. evaluated ten patients with TAK, and observed that AIx was higher in TAK compared to healthy controls, which also was confirmed in our study (Ng et al., 2006). Pulse wave velocity (PWV) is the most widely used method that measures the speed of arterial pressure waves traveling along large arteries. Neto et al. evaluated PWV in 27 female patients with TAK, and showed that patients with TAK had increased PWV compared to healthy controls (Salles Rosa Neto et al., 2014). Watanabe et al. has recently made a systematic review of fifteen studies with focus on pulse wave velocity (six studies), IMT (seven studies) and flow-mediated dilation (two studies) in TAK. They concluded that TAK patients demonstrated affected values in all these measured modalities. However, all included studies had a small number of patients, and none of the studies investigated the change in PWV, IMT and FMD during follow-up (Watanabe et al., 2021).

Cerebrovascular reserve capacity was not different in TAK compared to healthy controls, neither in those with nor without significant carotid or central artery stenosis/occlusion.

Diastolic velocity of MCA was lower in patients with TAK indicating a more high-resistant flow profile in TAK. This finding could be explained by increased arterial stiffness. Prior studies on TAK in this field are scarce. Six patients with TAK were compared to controls and no difference was detected concerning BHI (Pektezel et al., 2021). Transcranial Doppler parameters did not differ in patients with occluded carotid artery compared to controls, which is in line with our observations. A potential explanation could be a protected cerebral microcirculation in patients with TAK, unlike patients with atherosclerosis where BHI is impaired (Silvestrini et al., 2000).

Cantú et al. observed intracranial hemodynamic changes with dampened flow pattern and low flow pulsatility in ten patients with TAK with bilaterally extracranial stenosis, while patients with unilaterally stenosis showed no hemodynamic changes (Cantú et al., 2000). In our study only two patients had bilaterally extracranial stenoses, which may explain our normal BHI findings.

AIx75 and BHI did not correlate to hypertension, treatment, or any other background factors. However, our patient group is small and does not allow conclusions regarding influence of epidemiological factors as pinpointed above.

Ultrasound evaluation of IMT in subjects with TAK showed increased IMT in at least one vessel area. Patients with stenosis or occlusion in subclavian and/or axillary arteries are more difficult to evaluate regarding IMT, explaining missing data in 12–35% of these arteries and significant higher IMT only in CCA of TAK patients. In our group of TAK patients, only eight patients had small visible plaques, suggesting that most stenoses and occlusions are due to the inflammatory disease and not atherosclerosis.

The main limitation of our study was the small size of the patient group, reflecting the rarity of TAK in Scandinavia. Nevertheless, the included number of patients were comparable with many other studies investigating different aspects of the disease. Since the described vascular methods are to some degree operator dependent, a potential inter-operator dependent affection was eliminated with only one operator performing all methods.

The fiber-optic method used for evaluation of microcirculation could be difficult to compare with established methods as the way of analyze is newly developed. However, our method has been validated for various diseases and in large population studies (Jonasson et al., 2020). Test-retest variability was not possible to study for the microcirculation method as the hyperemic phase could affect a second measure.

## Conclusion

To conclude, we show that in addition to ultrasound evaluation of multiple arterial areas, measurements of microcirculation and pulse wave analysis, can add information regarding the total burden of vascular hemodynamic affection in TAK. Impaired microcirculation in the skin as reflected by OxyP was observed in TAK. This method for evaluation of microcirculation has previously not been used in patients with TAK. Impaired AIx75 was seen in TAK implying increased arterial stiffness.

Our results suggest that microcirculation and arterial stiffness in TAK are affected regardless of significant stenosis/occlusion of upstream arteries or not. Furthermore, preserved cerebrovascular hemodynamics in TAK and signs of increased arterial stiffness in the brain were detected. The value of these findings for prediction of future cardiovascular events needs to be clarified in further studies.

## Data Availability

The original contributions presented in the study are included in the article/Supplementary Material further inquiries can be directed to the corresponding author.

## Glossary

ACE: Angiotensin-converting enzyme
ACR: American college of rheumatology
AIx: Augmentation Index
AIx75: Augmentation Index normalized to heart rate 75
ARB: Angiotensin II receptor blocker
AXA: Axillar artery
BHI: Breath hold index
BMI: Body mass index
CABG: Coronary artery bypass graft surgery
CCA: Common carotid artery
CRP: C-reactive protein
CTA: Computed tomography angiography
CVR: Cerebrovascular reserve capacity
DRS: Diffuse reflectance spectroscopy
ECST: European carotid surgery trial
EDV: End diastolic velocity
FMD: Flow mediated dilation
hsCRP: High-sensitive C-reactive protein
HDL: High-density lipoprotein
ICA: Internal carotid artery
IMT: Intima-media thickness
IL-6: Interleukin-6
LDF: Laser Doppler flowmetry
LDL: Low-density lipoprotein
MFV: Mean flow velocities
MCA: Middle cerebral artery
MRI: Magnetic resonance imaging
OxyP: Peak oxygen saturation
PCI: Percutaneous coronary intervention
PSV: Peak systolic velocity
PWA: Pulse wave analysis
PWV: Pulse wave velocity
PORH: Post occlusive reactive hyperemia
ScA: Subclavian artery
SCAPIS: Swedish cardiopulmonary bioImage study
TAK: Takayasu Arteritis
TCD: Transcranial Doppler
TIA: Transient ischemic attacks
US: Ultrasound
