# Supplementary material for: Affected Microcirculation and Vascular Hemodynamics in Takayasu Arteritis
Source: Front Physiol. 2022 Jul 5;13:926940. doi: 10.3389/fphys.2022.926940 (PMC9294362; doi:10.3389/fphys.2022.926940)
Supplement: Supplementary file 1 [file Table1.DOCX]

|  |  |  |  |  |
| --- | --- | --- | --- | --- |
| **Patient** | **Sex** | **Age (Years)** | **Disease duration (Years)** | **Arteries involved** |
| 1 | F | 41 | 13 | Brachiocephalic trunc, ScA bil |
| 2 | F | 48 | 14 | Aortic arch, Brachiocephalic trunc, ScA bil |
| 3 | F | 55 | 26 | Brachiocephalic trunc, ScA bil, AxA R CCA bil |
| 4 | F | 58 | 40 | Brachiocephalic trunc, ScA bil, AxA bil, CCA bil |
| 5 | F | 50 | 2 | CCA bil |
| 6 | F | 33 | 1 | Brachiocephalic trunc, ScA bil, AxA L, CCA bil |
| 7 | F | 28 | 4 | ScA R, CCA L |
| 8 | F | 30 | 9 | ScA bil, CCA bil |
| 9 | M | 20 | 0 | Aortic arch, Brachiocephalic trunc, ScA L, CCA L |
| 10 | F | 22 | 3 | AxA L |
| 11 | F | 34 | 6 | Brachiocephalic trunc, ScA bil, AxA bil, CCA bil, ICA L |
| 12 | F | 61 | 32 | Brachiocephalic trunc, ScA L, AxA bil, CCA bil |
| 13 | F | 40 | 7 | Aortic arch, Brachiocephalic trunc |
| 14 | F | 53 | 5 | Aortic arch, Brachiocephalic trunc, ScA bil, Ax bil, CCA bil, Abdominal aorta, RA bil |
| 15 | M | 63 | 13 | ScA R, CCA bil |
| 16 | F | 21 | 2 | CCA bil, RA bil |
| 17 | M | 52 | 33 | Brachiocephalic trunc, ScA R, CCA R |
|  |  |  |  |  |
| **Supplementary table 1.** Arterial involvement in the Takayasu patients. F, Female; M, Male; ScA, Subclavian artery; AxA, Axillary artery; CCA, Common Carotid Artery; | | | | |
| ICA, Internal Carotid Artery; RA, Renal Artery; R, Right; L, Left; Bil, Bilateral | | | | |
